# A putative novel protein, DEPDC1B, is overexpressed in oral cancer patients, and enhanced anchorage-independent growth in oral cancer cells that is mediated by Rac1 and ERK

**DOI:** 10.1186/s12929-014-0067-1

**Published:** 2014-08-05

**Authors:** Ying-Fang Su, Chi-Yen Liang, Chih-Yang Huang, Chih-Yu Peng, Claire Chiyu Chen, Ming-Cheng Lin, Rong-Kai Lin, Wei-Wen Lin, Ming-Yung Chou, Pao-Hsin Liao, Jaw-Ji Yang

**Affiliations:** 1 Institute of Medicine, School of Dentistry, Taichung, 402, Taiwan; 2 Chung-Shan Medical University, School of Dentistry, Taichung, 402, Taiwan; 3 Department of Stomatology, Chung-Shan Medical University Hospital, Taichung, 402, Taiwan; 4 Chiayi Christian Hospital, Chiayi, 600, Taiwan; 5 Graduate Institute of Chinese Medical Science, Graduate Institute of Basic Medical Science, Taichung, 404, Taiwan; 6 China Medical University, Taichung, 404, Taiwan; 7 Department of Health and Nutrition Biotechnology, Asia University, Taichung, 413, Taiwan; 8 Department of Chemistry and Biochemistry, University of California at Los Angeles, Cardiovascular Center, Los Angeles 90095, CA, USA; 9 Veterans General Hospital, Taichung 407, Taiwan

**Keywords:** Anchorage-independent growth, Oral cancer, Extracellular-signal-regulated kinases, Rac1, DEPDC1B

## Abstract

**Background:**

The DEP domain is a globular domain containing approximately 90 amino acids, which was first discovered in 3 proteins: *Drosophila* disheveled, *Caenorhabditis elegans* EGL-10, and mammalian Pleckstrin; hence the term, DEP. DEPDC1B is categorized as a potential Rho GTPase-activating protein. The function of the DEP domain in signal transduction pathways is not fully understood. The DEPDC1B protein exhibits the characteristic features of a signaling protein, and contains 2 conserved domains (DEP and RhoGAP) that are involved in Rho GTPase signaling. Small GTPases, such as Rac, CDC42, and Rho, regulate a multitude of cell events, including cell motility, growth, differentiation, cytoskeletal reorganization and cell cycle progression.

**Results:**

In this study, we found that it was a guanine nucleotide exchange factor and induced both cell migration in a cultured embryonic fibroblast cell line and cell invasion in cancer cell lines; moreover, it was observed to promote anchorage-independent growth in oral cancer cells. We also demonstrated that DEPDC1B plays a role in regulating Rac1 translocated onto cell membranes, suggesting that DEPDC1B exerts a biological function by regulating Rac1. We examined oral cancer tissue; 6 out of 7 oral cancer tissue test samples overexpressed DEPDC1B proteins, compared with normal adjacent tissue.

**Conclusions:**

DEPDC1B was a guanine nucleotide exchange factor and induced both cell migration in a cultured embryonic fibroblast cell line and cell invasion in cancer cell lines; moreover, it was observed to promote anchorage-independent growth in oral cancer cells. We also demonstrated that DEPDC1B exerts a biological function by regulating Rac1. We found that oral cancer samples overexpressed DEPDC1B proteins, compared with normal adjacent tissue. Suggest that DEPDC1B plays a role in the development of oral cancer. We revealed that proliferation was linked to a novel DEPDC1B-Rac1-ERK1/2 signaling axis in oral cancer cell lines.

## Background

The DEP domain is a globular domain containing approximately 90 amino acids, which was first discovered in 3 proteins: *Drosophila* disheveled, *Caenorhabditis elegans* EGL-10, and mammalian Pleckstrin; hence the term, DEP [1]–[3]. The DEP domain was observed to play a function in mediating membrane localization and regulating a broad range of cellular functions [4], from the determination of cell polarity to highly specialized signals in photoreceptors of the retina. The DEP domain contains a cluster of basic residues that enable it to interact with negatively charged phospholipids located in membranes; this may be required for Wnt signaling [5]. Moreover, DEP domain proteins enable direct interaction with G protein-coupled receptors and mediated GPCR signaling pathways [6],[7].

The function of the DEP domain in signal transduction pathways is not fully understood. The DEPDC1B protein exhibits the characteristic features of a signaling protein, and contains 2 conserved domains (DEP and RhoGAP) that are involved in Rho GTPase signaling. Small GTPases, such as Rac, CDC42, and Rho, regulate a multitude of cell events, including cell motility, growth, differentiation, cytoskeletal reorganization and cell cycle progression [8]. Rac and Cdc42 activation have been linked to the formation of lamellipodia and filopodia, respectively, whereas Rho protein activation has been associated with the formation of actin stress fibers [9],[10]. Among these GTPases, Rac1 activity has been implicated in tumorigenesis in various tissues [11],[12]. Rac1 activation increases cell proliferation, and alters cell migration and mitogen-activated protein kinase (MAPK) signaling. MAPK signaling, including ERK, p38 and JNK, is involved in a variety of cellular functions, such as growth, proliferation, differentiation, and apoptosis [13]. Of the signaling pathways, ERK has been studied the most in-depth. ERK activation induces numerous biological responses that involve cell proliferation, angiogenesis, and differentiation [14].

We found that DEPDC1B was highly expressed in oral cancer tissue, compared with normal adjacent tissue. The overexpression of DEPDC1B in cells promotes cell migration and induces cell invasion in cancer cell lines. The effects of DEPDC1B on both migration and invasion are mediated by Rac1. DEPDC1B affects the loading and augmentation of ERK1/2 activity by Rac1 GTP, which subsequently causes colony formation in oral cancer cells. We revealed a novel DEPDC1B-Rac1-ERK1/2 signaling axis in the development of oral cancer cell lines. The identification of molecular networks using DEPDC1 in this study could be useful for the future discovery of novel therapeutic targets and diagnostic markers to treat cancers.

## Methods

### Northern blot analysis

A human tissue blot (Clontech) was hybridized with a probe corresponding to DEPDC1B full-length cDNA and labeled using an NEBlot random labeling kit (New England BioLabs) in the presence of [α-^32^P] dCTP. The blot was washed with SSC/SDS solution (sodium chloride, sodium citrate/SDS) before autoradiography.

#### Immunoprecipitation and western blot analysis

Cell lysates were prepared in IP buffer (40 mM Tris–HCl [pH 7.5], 1% NP40, 150 mM NaCl, 5 mM EGTA, 1 mM DTT, 1 mM PMSF, 20 mM NaF, proteinase inhibitors, and 1 mM sodium vanadate). Cell extracts (600 μg) were incubated with 5 μg of primary antibody for 6 h at 4°C, mixed with 20 μL of protein-A sepharose suspension, and incubated for an additional hour. Immunoprecipitates were collected by centrifugation, washed 3 times with IP buffer plus 0.5% deoxycholate, and 5 times with IP buffer alone, before being subjected to SDS-PAGE. Immunoblot analysis was performed with specific antibodies against, Rho, CDC42, and Rac1 (Cell Biolab, Inc). Cell-expressing DEPDC1B or the empty vector were harvested in lysis buffer (50 mM Tris–HCl, pH 8.0/250 mM NaCl/1% NP-40, 2 mM EDTA) containing 1 mM PMSF, 10 ng/mL leupeptin, 50 mM NaF, and 1 mM sodium orthovanadate. Total proteins were then separated on SDS-PAGE then Immunoblot analysis was performed with specific antibodies against MAPKs, P38, pp38, pJNK, ppJNK, pERK, and ppERK (Cell Signaling Technology) and specific protein bands were visualized using an ECL chemiluminescent detection system (Amersham).

#### Wound-healing assay

Cells seeded on 10-cm plates were cultured to confluency. They were then scratched with a 200-μL pipette tip and incubated in DMEM supplemented with 10% FBS. Images were taken at 17 h with a Zeiss Axiovert 200 microscope.

#### Membrane and cytosol fractionation

Cells were cultured with 1 μg/mL doxycycline for 48 h and then treated with a lysis buffer (20 mM Tris–HCl, pH 7.5, 100 mM NaCl, 5 mM EDTA, 2 mM PMSF, 1× protease inhibitor) at 4°C for 30 min. The samples were centrifuged at 500 × g at 4°C for 10 min, and the pellets were dissolved in lysis buffer plus 0.1% (w/v) Triton X-100 for the membrane fractions. The supernatants were recentrifuged at 15 000 rpm at 4°C for 20 min, and the supernatants were saved as cytosolic fractions.

### Cell migration assay

A migration assay using a Boyden chamber was performed by filling the bottom well of the chamber with DMEM medium containing 10% FBS. Wells were covered with polyvinylpyrrolidone-free polycarbonate membranes with 8-μm pores (Neuro Probes, Inc.), and 1500 cells/well in serum-free DMEM were added to the top chamber. The Boyden chamber was incubated for 24 h at 37°C to allow the possible migration of cells through the membrane into the bottom chamber. Membranes were stained using Giemsa stain. The cells in the bottom chamber were counted using a grid fitted into the eyepiece of a phase contrast microscope.

Experimental research reported in the manuscript has been performed with the approval of the Institutional Review Board of Taichung Veterans General Hospital (IRBTCVGH No: 950727/C06134).

## Results

### Tissue distribution of DEPDC1B mRNA

**Figure 1:**
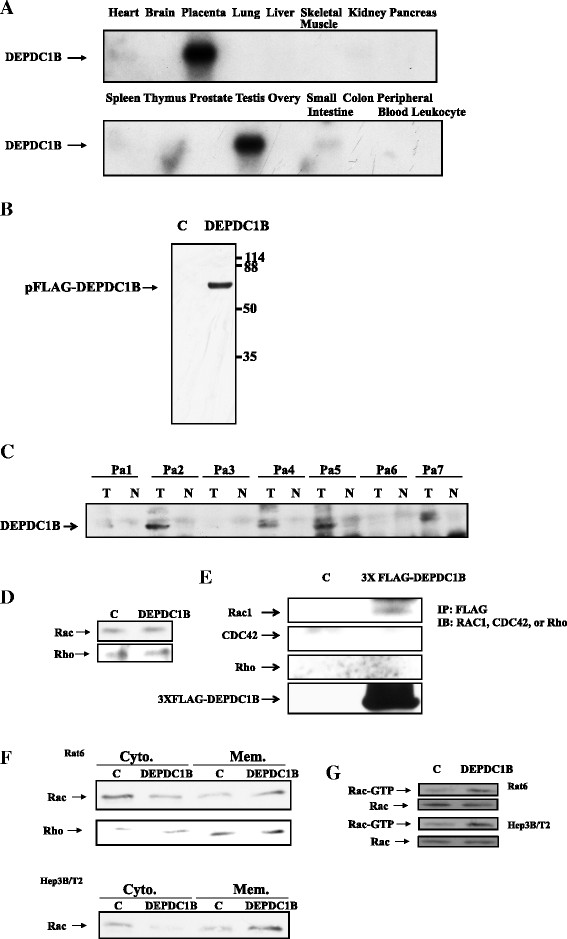
**Expression patterns of DEPDC1B in normal tissues and tumor cells and effects of DEPDC1B on Rho GTPase expression and the subcellular localization of Rac1. (A)** Expression of DEPDC1B mRNA as presented by northern blot analysis of various human tissues, by using DEPDC1B cDNA as a probe. A human multiple tissue northern blot containing poly (A)^+^ RNA was probed with full-length DEPDC1B DNA. **(B)** FLAG epitope-tagged forms of DEPDC1B were transiently expressed in 293 T cells, and FLAG-DEPDC1B proteins were detected by using antiFLAG antibodies with a molecular weight of approximately 58 kDa. **(C)** DEPDC1B protein was abundantly expressed in oral cancer tissue (T) compared with cognate normal tissue (N). **(D)** Rho, and Rac1 were detected by western blotting of cell lysates from Rat6 cells stably expressing DEPDC1B. **(E)** 3X FLAG epitope-tagged forms of DEPDC1B were transiently expressed in 293 T. Anti-FLAG antibodies were used for immunoprecipitation. Anti-Rho, −CDC42, and -Rac1 antibodies were used for western blotting. **(F)** Membrane (Mem.) and cytosolic (Cyto.) fractions from Rat6 or Hep3B/T2 control (C) and DEPDC1B-expressing cells were analyzed using immunoblotting for Rac1 and RhoGDIβ. **(G)** GTP loading in Rac1 was determined and a PAK PBD binding assay was used to analyze in Rat6 or Hep3B/T2 cells that stably expressed DEPDC1B.

To ascertain the expression pattern of the DEPDC1B gene, we studied the endogenous expression of DEPDC1B mRNA in various human tissues. Northern blot analysis of the tissues demonstrated that the mRNA for DEPDC1B was 4.6 kb. DEPDC1B gene expression was only detected in a few tissues, and was abundant in the placenta and testis, and relatively scarce in the heart and small intestine (Figure 1A). The open reading frame of DEPDC1B encodes a putative polypeptide of 530 amino acids, with a calculated molecular mass of 58.3 kDa. To ascertain the expression and molecular weight of DEPDC1B, 293 T cells were transfected with plasmids expressing a FLAG-tagged DEPDC1B construct. The expressed proteins were determined using western blot analysis, using an antibody specific for FLAG. A band at a molecular weight of 59 kDa was detected (Figure 1B). To evaluate the expression level of DEPDC1B protein in oral cancer tissue, we performed an immunoblotting assay using human oral cancer tissue. Among the 7 oral cancer tissues that were evaluated, 6 overexpressed DEPDC1B proteins in comparison with normal adjacent tissue (Figure 1C). The data suggested that DEPDC1B may play a role in the development of oral cancer.

#### Expression of DEPDC1B modulates Rac1 cellular localization in rat embryonic fibroblasts

DEPDC1B encodes a protein that may function as a Rho GTPase-activating protein (GAP), according to its intrinsic primary protein sequences; therefore, it may play a role in regulating Rho GTPase activity. Many studies have indicated that Rho GTPases act as molecular switches by cycling between the inactive GDP-bound form located in the cytoplasm and an active membrane-associated GTP-bound form. The activities of Rho family proteins are regulated by various proteins, such as guanine nucleotide exchange factors (GEFs), GAPs, and GDP-dissociation inhibitors [15]. We then characterized the biological effects of DEPDC1B on cultured cell systems. We generated stable rat embryonic fibroblast, Rat6, and hepatoma Hep3B cells that expressed DEPDC1B under tetracycline-responsive transactivator control. In this system, the addition of the tetracycline analog doxycycline induced the expression of DEPDC1B. We then sought to determine whether DEPDC1B stimulated the expression or activities of these GTPases in cultured cells by using western blotting. Total Rac1 and Rho levels remained the same in DEPDC1B-overexpressing cells (Figure 1D). We therefore concluded that DEPDC1B might not regulate the expression of these Rho GTPases. Because DEPDC1B encodes a putative protein that could function as a regulator or be physically associated with Rho GTPases, we sought to determine whether DEPDC1B was able to bind to these Rho GTPases. This interaction was investigated using in vivo coprecipitation. 293 T cells were transfected with plasmids that expressed a FLAG-tagged DEPDC1B. Protein complexes were immunoprecipitated using antiFLAG antibodies. Coprecipitated Rho GTPase proteins were detected by Rac1, CDC42, or RhoA antibodies in immunoblotting analysis. As illustrated in Figure 1E, Rac1 protein was detected in the FLAG-DEPDC1B immunoprecipitated complexes, indicating that DEPDC1B proteins may have physically interacted with the Rac1 protein. Therefore, DEPDC1B might be a potential RhoGEF and contribute to the activation of Rac1.

To further address the question of whether DEPDC1B influences Rho GTPase activity, a detergent-insoluble membrane fraction was prepared from DEPDC1B-overexpressing cells, and membrane-associated GTPases were determined using western blotting. The level of membrane-associated Rac1 increased in DEPDC1B-overexpressing cells, whereas the cytosolic form of Rac1 decreased (Figure 1F). The membrane-associated and cytosolic forms of RhoA remained unchanged in DEPDC1B-expressing cells compared with parental cells. Therefore, overexpression of DEPDC1B in cells increased the level of membrane-associated Rac1, which was dissociated from expression levels of Rac1 protein. We detected the amount of GTP-bound Rac1 in Rat6 and Hep3B DEPDC1B-expressing cells, and determined that DEPDC1B stimulated GTP loading in Rac1 (Figure 1G). The expression of DEPDC1B in both Rat6 or Hep3B cells increased the level of membrane-associated Rac1 and GTP loading in Rac1.

#### Rac1 controls cell adhesion and motility

**Figure 2:**
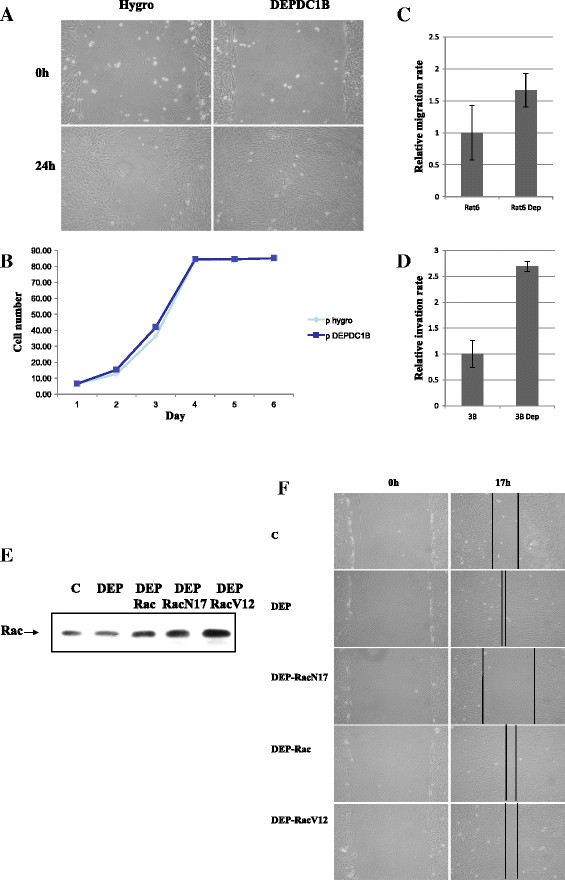
**DEPDC1B effects on the induction of cell migration in Rat6 cells and tumor cell invasion was Rac1-dependent. (A)** Wound-healing assay. Rat6 cells and Rat6 cells ectopically expressing DEPDC1B were seeded onto plates. After reaching confluency, the cell layer was wounded with a 200-μL pipette tip and incubated for 24 h. **(B)** Growth curves of Rat6 cells with and without expression of DEPDC1B. Stable Rat6 cell lines were seeded at an original density of 5000 cells per 6 well dishes, and cell numbers were counted at the indicated time. **(C)** DEPDC1B enhanced cell migration in Rat6 cells. Migration was measured by quantifying the number of cells that had migrated to the lower chamber of a Boyden chamber after 16 h. **(D)** DEPDC1B enhanced cell invasion in Hep3B/T2 cells. Invasion was measured by quantifying the number of cells that had migrated to the lower chamber of a Boyden chamber after 16 h. **(E)** Control Rat6 and Rat6 DEPDC1B-expressing cells stably transfected with Rac1, Rac1N17, or Rac1V12 were grown in 10% fetal bovine serum; Rac1 expression levels were determined. **(F)** Wound-healing assay. The cell lines were seeded on plates, as previously described. After reaching confluency, the cell layer was wounded with a 200-μL pipette tip and incubated for 17 h.

Our data suggested that DEPDC1B was able to bind to and regulate Rac1 activities. To test the effect of DEPDC1B on cell migration, confluent monolayers of cells that stably expressed DEPDC1B were scrape-wounded with a sterile plastic pipette, and the migration of cells into the wound was monitored. The DEPDC1B-expressing cells closed the wound area faster than the control cells (Figure 2A). To determine whether DEPDC1B played a role in the induction of cell proliferation, contributing to faster wound healing, we examined the growth rate of cells expressing DEPDC1B and control cells. We found no substantial difference between the growth rates of DEPDC1B-expressing cells and control cells (Figure 2B). DEPDC1B-regulated cell migration was not mediated through increased cell cycle progression. We used migration assays to confirm the role of DEPDC1B in cell migration. DEPDC1B-expressing cells and parental cells were seeded on a porous filter in the upper chamber of a transwell. The migration through the filter pores of Rat6 cells expressing DEPDC1B was increased compared with parental cells (Figure 2C). To further confirm the role of DEPDC1B in cell invasion, DEPDC1B-expressing hepatoma cells and parental cells were seeded on a porous filter in the upper chamber of a transwell, with matrigel present on top of the filter. DEPDC1B-expressing hepatoma cells exhibited a substantially increased invasion rate compared with the parental cells (Figure 2D). The data suggested that when DEPDC1B was expressed in cells, cellular motility was stimulated and invasion ability in tumor cells increased.

To test whether the effect of DEPDC1B on cell migration was Rac1-dependent, DEPDC1B cells were transfected with plasmids harboring wild-type Rac1 (Rac), dominant-negative Rac1 (RacN17), and constitutively active Rac1 (RacV12) (Figure 2E). Confluent monolayers of cells stably expressing DEPDC1B, DEPDC1B-Rac1, or DEPDC1B − Rac1N17, DEPDC1B − Rac1V12 were scrape-wounded with a sterile plastic pipette, and the migration of cells into the wound was monitored. As previously demonstrated, DEPDC1B-expressing cells closed the wound area faster than the control cells (Figure 2F). The DEPDC1B-Rac1N17 cells migrated more slowly than the DEPDC1B, DEPDC1B-Rac1 and DEPDC1B-Rac1V12 cells (Figure 2F), suggesting that Rac1 plays a key role in mediating cell migration in DEPDC1B-expressing cells. These findings indicated that DEPDC1B-induced cell migration in Rat6 cells was mediated through the increase of membrane-associated Rac1 and stimulation of GTP loading in Rac1. This suggests that DEPDC1B stimulated cell migration that was mediated through Rac1.

Because the small GTPase Rac1 acted as a bridge for DEPDC1B to induce cellular functions, we tested the role of DEPDC1B to see whether it potentiated tumor formation in an oral cancer cell line, KB. We then measured the overexpression of DEPDC1B in KB cells. These cells were tested for their ability to grow in soft agar.

#### DEPDC1B potentiates colony formation in KB cells

**Figure 3:**
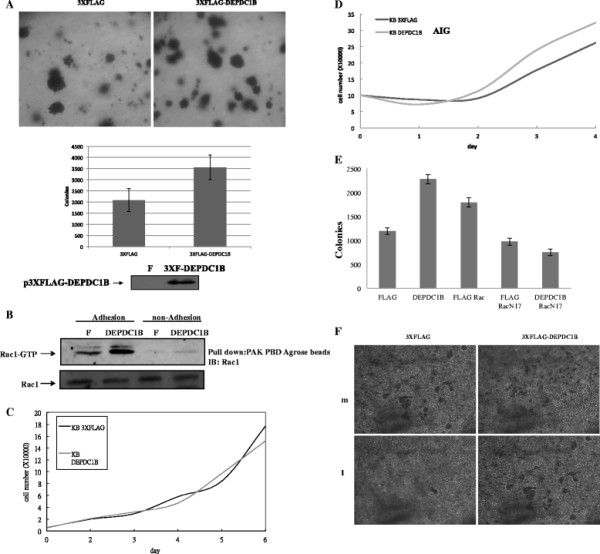
**Soft agar colony formation by KB and KB-DEPDC1B cells and DEPDC1B-enhanced anchorage-independent growth was Rac1-dependent. (A)** The upper panel presents relative colony size and the lower panel presents the quantitation of the results. The lower panel shows the ectopic DEPDC1B expression levels. **(B)** GTP loading in Rac1 was determined and the PAK PBD binding assay was used to analyze in KB or DEPDC1B-expressed KB cells in adherent and nonadherent cultured conditions. **(C)** Stable KB cell lines were seeded at an original density of 5000 cells per 6 well dishes, and the cell number was counted at the indicated time. **(D)** Stable KB cell lines were seeded at an original amount of 10 000 cells in a 50-mL conical tube with methylcellulose; cell numbers were counted at the indicated time. **(E)** Control KB and KB DEPDC1B-expressing cells stably transfected with Rac1 or Rac1N17were grown in soft agar. **(F)** DEPDC1B enhanced cell migration (M) and invasion (I) in KB cells.

The overexpressed DEPDC1B protein in KB cells potentiated to colony formation by approximately 1.7 fold, compared with vector-transfected parental cells (Figure 3A). The data suggested that DEPDC1B proteins stimulated anchorage-independent growth in an oral cancer cell line. To confirm the expression of DEPDC1B in such oral cells, we employed a PAK PBD pull-down assay to test whether the DEPDC1B expressed in oral cancer cells induced GTP loading in Rac1 proteins. Figure 3B illustrates that DEPDC1B proteins increased GTP loading in Rac1 proteins in oral cancer cells when the cells were growing in adherent or nonadherent conditions. These results indicated that DEPDC1B was a potential GEP in all tested cells, including Rat6, Hep3B, and KB cells.

To determine whether DEPDC1B played a role in the induction of cell proliferation in oral cancer cells, we examined the growth rate when cells were both with and without the DEPDC1B expression, in growth conditions of adhesion and non-adhesion (Figure 3C and D). We found that DEPDC1B-expressed cells exhibited a higher growth rate than the control mock-transfected cells in anchorage-independent conditions, whereas there was no substantial change to adherent conditions. The results indicated that DEPDC1B was able to promote cancer cell proliferation in nonadherent conditions. Moreover, the overexpression of DEPDC1B in cells can trigger Rac1 activation. We then tested whether the ability of DEPDC1B to promote growth was mediated through Rac1. The anchorage-independent growth ability in soft agar of the mutant Rac1 (Rac1 N17) coexpressed with DEPDC1B in these cells and oral cancer cells was examined and compared with DEPDC1B cells. We confirmed that the cell proliferation ability induced by DEPDC1B was abolished with the coexpressed Rac1 N17 proteins in oral cancer cells (Figure 3E). The results indicated that the biological function of DEPDC1B proteins to induce cell proliferation was mediated through Rac1 proteins.

We used migration and invasion assays to confirm the role of DEPDC1B in oral cancer cell migration and invasion. DEPDC1B-expressing KB cells and parental cells were seeded on a porous filter in the upper chamber of a transwell. The migration and invasion through the filter pores of KB cells expressing DEPDC1B was increased compared with parental cells (Figure 3F). The data suggested that when DEPDC1B was expressed in oral cancer cells, cellular motility and invasion ability was stimulated.

#### DEPDC1B induces cell growth through a DEPDC1B-Rac1-ERK1/2 signaling

**Figure 4:**
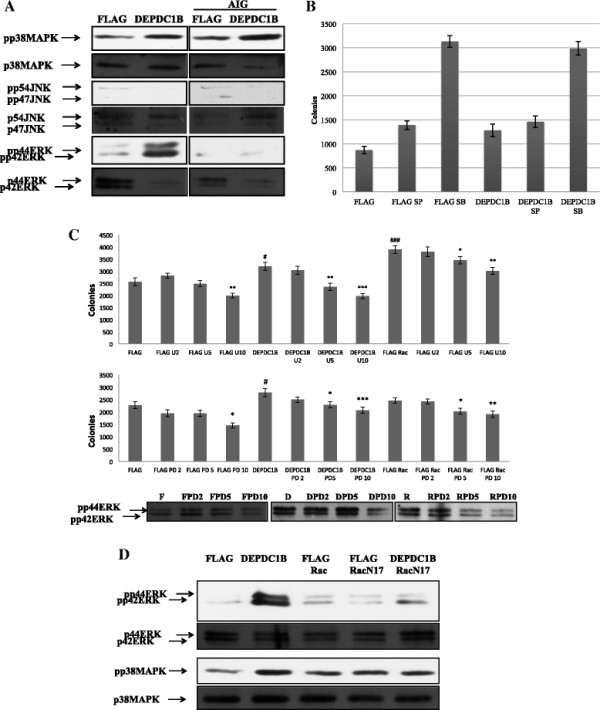
**MAPK activity followed KB cells transfected with DEPDC1B expression plasmids. (A)** Following the transfection of KB cells with DEPDC1B expression plasmids in adherent and nonadherent culture conditions (AIG), ERK and p38 MAPK activities were induced, whereas JNK activities were suppressed (AIG). 50 μg of total cellular proteins were analyzed using western blotting and probed with specific antibodies. **(B)** DEPDC1B induced soft agar colony formation not mediated through JNK or p38. KB and KB-DEPDC1B cells were inoculated into soft agar medium containing different concentrations of either SB203580 or SP600125 (10 μM). **(C)** DEPDC1B induced soft agar colony formation mediated through ERK. KB, KB-DEPDC1B, and KB-DEPDC1B-Rac1 cells were inoculated into soft agar medium containing different concentrations of either U0126 or PD98059 (0, 2, 5, 10 μM). The results are presented as mean ± S.E. *, P < 0.05, **, 0.001 ≤ P < 0.01, ***, P < 0.001. The lower panel indicates the concentration of drug to block activity of ERK and at the concentration of 10 μM PD98059 has the most significant to block ERK activity to 50%. **(D)** DEPDC1B-enhanced ERK activation was Rac1-dependent. Stable KB cell lines were seeded in a 50-mL conical tube with methylcellulose. Cell lysates were harvested 72 h later. Subsequently, 50 μg of total cellular proteins were analyzed using western blotting and probed with specific antibodies.

To investigate whether DEPDC1B regulated additional signal transduction pathways, we tested DEPDC1B proteins on the activation of MAPK pathways (including p38 MAPK, JNK, and ERK). For all the MAPK pathways tested, we observed that the expression of DEPDC1B proteins in oral cancer cells induced p38 MAPK and ERK activity; however, it suppressed JNK activation (Figure 4A). To determine which MAPK pathway mediated growth induced by DEPDC1B, we employed kinase-specific inhibitors (SB203580, SP600125, U0126, and PD98059) to determine whether the anchorage-independent growth rates were influenced by the treatment of these inhibitors. SP600125-treated DEPDC1B-expressed or parental cells grew as many colonies as cells that were not treated using this inhibitor (Figure 4B). This result indicated that JNK activities were not involved in the promotion of growth in DEPDC1B-expressed cells, whereas cells treated with SB203580 grew more colonies than the cells that were not treated using this inhibitor (Figure 4B). Because p38 MAPK activities were induced by the expression of DEPDC1B in cells, it was assumed that increasing p38 MAPK activity correlates with the promotion of anchorage-independent growth induced by DEPDC1B. However, treatment with p38-MAPK-specific inhibitors increased colony formation in DEPDC1B-expressed cells, suggesting that p38 MAPK caused the opposite effect on growth-promoting properties. Neither p38 MAPK nor JNK activity mediated the promotion of anchorage-independent growth induced by DEPDC1B. Whereas all cells (parental, DEPDC1B-expressed, and Rac-expressed) were treated using U0126 or PD98059, the anchorage-independent growth induced by DEPDC1B was suppressed by both inhibitors in a dose-dependent manner (Figure 4C). The results suggested that ERK activity mediates growth promotion induced by the expression of DEPDC1B and Rac in oral cancer cells. To determine whether DEPDC1B activation of ERK was mediated through Rac, we cotransfected DEPDC1B with dominant-negative Rac (Rac N17), and found that ERK activity induced by DEPDC1B were influenced by the expression of Rac N17 proteins. This data indicated that Rac acted as an intermediate molecule downstream of DEPDC1B for ERK activity (Figure 4D). We found that DEPDC1B was a growth-promoting protein that activated Rac and then triggered ERK activity to enhance anchorage-independent growth in oral cancer cells.

## Discussion

In this paper, we report the identification and characterization of a novel gene, DEPDC1B (DEP domain-containing 1B), which was found to be considerably expressed in placenta and the testis, but less so in the heart and small intestine. The northern blotting analysis results indicated that the gene was not detectable in other kinds of human tissue. DEP domain-containing proteins regulate numerous cellular functions; DEP domain-containing proteins include signaling proteins, including disheveled, EGL-10 and Pleckstrin. DEPDC1B harbors a DEP domain. DEP domains can be found in Rho family GEFs, as well as in certain GAPs; however, its biological role in cells has not been investigated. To elucidate the biological role of DEPDC1B, we cloned DEPDC1B cDNA. This cDNA was then subcloned into mammalian expression vectors. We found that DEPDC1B regulated Rac1 activities by increasing GTP loading in Rac1 did not affect Rho A activities in either normal or cancer cells. In an immunoprecipitation experiment, we found that DEPDC1B was able to physically interact with Rac1, but not Rho A or CDC 42. We demonstrated that DEPDC1B proteins function as GEFs and specifically activate Rac1.

Rac1 GTPase participates in regulating the migration, invasion, transformation, growth, and survival of tumor cells [16]. Because DEPDC1B acts as an upstream regulator for Rac1, testing the mobility of DEPDC1B-expressing cells can be beneficial. DEPDC1B plays a regulatory role by functioning as a GEF that activates Rac1 proteins and triggers migration in normal cells and invasion in tumor cells.

We described a finding that revealed DEPDC1B proteins were overexpressed in oral cancer tissue, compared with normal adjacent tissue, in 6 out of 7 patients. We then demonstrated that DEPDC1B proteins promoted anchorage-independent growth in the KB cultured oral cancer cell line. Our model suggested that DEPDC1B was a positive modulator of Rac1 in oral cancer cell lines cultured in both adherent and nonadherent conditions. By using genetic approaches, we provided evidence that DEPDC1B regulates anchorage-independent growth mediated through Rac1 in oral cancer cells. Furthermore, DEPDC1B potentiates anchorage-independent growth signals for the activation of ERK1/2, which critically mediates the functions of DEPDC1B. Our data revealed that DEPDC1B affected Rac1 GTP loading and augmented ERK1/2 activity, causing subsequent colony formation in oral cancer cells. We revealed that the proliferation was linked to the DEPDC1B-Rac1-ERK1/2 signaling axis in the oral cancer cell lines. DEPDC1B, acted as a potentially oncogenic protein in oral cancer patients, contributing to the sustained elevation of ERK1/2 activity throughout the stimulation of the GDP-GTP exchange in Rac1. ERK1/2 activity regulates cancer cell proliferation and is a crucial factor in cancer progression. Our results suggested a novel route by which DEPDC1B regulates Rac1 activation and modulates ERK1/2 activities, and offer an explanation for the mechanism by which DEPDC1b contributes to anchorage-independent growth in oral cancer cells.

## Conclusion

DEPDC1B was a guanine nucleotide exchange factor and induced both cell migration in a cultured embryonic fibroblast cell line and cell invasion in cancer cell lines; moreover, it was observed to promote anchorage-independent growth in oral cancer cells. We also demonstrated that DEPDC1B exerts a biological function by regulating Rac1. We found that oral cancer samples overexpressed DEPDC1B proteins, compared with normal adjacent tissue. Suggest that DEPDC1B plays a role in the development of oral cancer. We revealed that proliferation was linked to a novel DEPDC1B-Rac1-ERK1/2 signaling axis in oral cancer cell lines.

## Consent

Written informed consent was obtained from the patient for the publication of this report and any accompanying images.

## Competing interests

The authors declare that they have no competing interests.

## Authors’ contributions

Y-FS: Design and assembly data, C-YL: Conception, collection, and assembly of data, C-YH: Conception, collection, and assembly of data ,C-YP: Provision of study material or patients, CCC: experiment implementation, M-CL: Provision of study material, collection, and assembly of data, R-KL: Experiment implementation, W-WL: Provision of study material, collection and assembly of data, M-YC: Conception and design, financial support, P-HL and J-JY: Design, administrative support, manuscript writing, final approval of manuscript. All authors read and approved the final manuscript.
